# Supplementary material for: Functional Potential of Sweet Cherry Cultivars Grown in New Zealand: Effects of Processing on Nutritional and Bioactive Properties
Source: Foods. 2025 Oct 31;14(21):3749. doi: 10.3390/foods14213749 (PMC12610261; doi:10.3390/foods14213749)
Supplement: Supplementary file 1 [file foods-14-03749-s001.zip › foods-3906992-supplementary.pdf]

Table S1: The nutritional composition of six cherry cultivars (both fresh and processed) expressed as percentage dietary reference value per serve based on Food Standards Australia New Zealand (FSANZ) regulations (Food Standards Australia New Zealand 2018; Food Standards Australia New Zealand 2024) [35, 56].

		Kordia®		'Lapins'		Sweetheart®		Staccato®		'Bing'	'Rainier'
**Component**	**Reference value**	**Fresh**	**Processed**	**Fresh**	**Processed**	**Fresh**	**Processed**	**Fresh**	**Processed**	**Processed**	**Processed**
**Energy**	8700 kJ	5%	5%	5%	4%	5%	5%	5%	5%	5%	5%
**Fat, total**	70 g	1%	2%	1%	1%	1%	1%	1%	1%	1%	2%
**Protein**	50 g	3%	2%	2%	2%	3%	2%	2%	2%	3%	3%
**Total carbohydrate**	310 g	8%	8%	8%	7%	9%	8%	9%	8%	8%	7%
**Total sugars**	90 g	23%	23%	22%	20%	24%	22%	24%	23%	22%	21%
**Dietary fibre**	30 g	6%	6%	4%	6%	5%	6%	5%	6%	6%	6%
**Vitamin C**	40 mg	18%	18%	15%	15%	18%	20%	21%	21%	12%	14%
**Thiamin (B1)**	1.1 mg	4%	3%	3%	3%	4%	3%	4%	3%	3%	4%
**Riboflavin (B2)**	1.7 mg	4%	4%	3%	3%	4%	4%	4%	3%	3%	3%
**Niacin (B3)**	10 mg	1%	0%	1%	1%	1%	0%	1%	0%	2%	0%
**Vitamin B6**	1.6 mg	3%	3%	7%	7%	8%	7%	8%	8%	7%	6%
**Vitamin E, ATE**	10 mg	3%	3%	2%	2%	2%	3%	3%	3%	2%	2%
**Vitamin K**	80 µg	7%	7%	nd	nd	nd	nd	nd	nd	nd	nd
**Vitamin A, RE**	750 µg	0%	0%	1%	0%	1%	0%	0%	0%	0%	0%
**Calcium**	800 mg	2%	2%	2%	2%	2%	2%	0%	3%	2%	2%
**Magnesium**	320 mg	5%	5%	4%	5%	6%	5%	5%	5%	5%	5%
**Phosphorus**	1000 mg	4%	3%	3%	3%	4%	3%	3%	3%	3%	3%
**Iron**	12 mg	3%	2%	2%	3%	4%	3%	2%	3%	3%	2%
**Copper**	3 mg	6%	5%	10%	4%	7%	4%	4%	5%	4%	4%
**Manganese**	5 µg	2%	2%	2%	2%	2%	3%	2%	3%	4%	2%
**Zinc**	12 mg	1%	1%	2%	1%	2%	2%	1%	1%	1%	1%

Notes: ATE = alpha-tocopherol equivalents; RE = retinol equivalents; nd = not detected.

Table S2: A list of phenolic metabolites and classifications used in this study along with respective CAS* numbers.

Polyphenol Classification	Metabolite	CAS Number
Hydroxycinnamic acid	3,5-Dicaffeoylquinic acid	2450-53-5
Hydroxycinnamic acid	3-*p*-Coumaroyl quinic acid	1899-30-5
Hydroxycinnamic acid	Chlorogenic acid	327-97-9
Hydroxycinnamic acid	Ferulic acid	1135-24-6
Hydroxycinnamic acid	Neochlorogenic acid	202650-88-2
Hydroxycinnamic acid	*trans*-4-*p*-Coumaroyl quinic acid	1108200-72-1
Hydroxycinnamic acid	*trans*-5-*p*-Coumaroyl quinic acid	5746-55-4
Flavanol	Catechin	154-24-4
Flavanol	Epicatechin	490-49-0
Flavonol	Kaempferol 3-rutinoside	17650-84-9
Flavonol	Quercetin 3-galactoside	482-36-0
Flavonol	Quercetin 3-glucoside	482-35-9
Flavonol	Quercetin 3-rhamnoside	522-12-3
Flavonol	Quercetin 3-rutinoside	153-18-4
Flavanone	Sakuranetin	2957-21-3
Flavanone	Sakuranin	529-39-5
Procyanidin	Procyanidin B1	20315-25-7
Procyanidin	Procyanidin B2	29106-49-8
Procyanidin	Procyanidin B5	12798-57-1
Procyanidin	Procyanidin B7	12798-59-3
Anthocyanin	Cyanidin 3-glucoside	7084-24-4
Anthocyanin	Cyanidin 3-rutinoside	18719-76-1
Anthocyanin	Peonidin 3-rutinoside	218290-50-7

*CAS = Chemical Abstracts Service
